# Instant effects of peppermint essential oil on the physiological parameters and exercise performance

**Published:** 2014

**Authors:** Abbas Meamarbashi

**Affiliations:** 1 *Department of Physical Education and Sports Sciences, University of Mohaghegh Ardabili, Ardabil, I. R. Iran *

**Keywords:** *Isometric force*, *Peppermint*, *Reaction time*, *Spirometry*

## Abstract

**Objective:** Effect of peppermint on exercise performance was previously investigated but equivocal findings exist. This study aimed to investigate the effects of peppermint ingestion on the physiological parameters and exercise performance after 5 min and 1 h.

**Materials and Methods:** Thirty healthy male university students were randomly divided into experimental (n=15) and control (n=15) groups. Maximum isometric grip force, vertical and long jumps, spirometric parameters, visual and audio reaction times, blood pressure, heart rate, and breath rate were recorded three times: before, five minutes, and one hour after single dose oral administration of peppermint essential oil (50 µl). Data were analyzed using repeated measures ANOVA.

**Results:** Our results revealed significant improvement in all of the variables after oral administration of peppermint essential oil. Experimental group compared with control group showed an incremental and a significant increase in the grip force (36.1%), standing vertical jump (7.0%), and standing long jump (6.4%). Data obtained from the experimental group after five minutes exhibited a significant increase in the forced vital capacity in first second (FVC_1_)(35.1%), peak inspiratory flow rate (PIF) (66.4%), and peak expiratory flow rate (PEF) (65.1%), whereas after one hour, only PIF shown a significant increase as compare with the baseline and control group. At both times, visual and audio reaction times were significantly decreased. Physiological parameters were also significantly improved after five minutes. A considerable enhancement in the grip force, spiromery, and other parameters were the important findings of this study.

**Conclusion** **:** An improvement in the spirometric measurements (FVC_1_, PEF, and PIF) might be due to the peppermint effects on the bronchial smooth muscle tonicity with or without affecting the lung surfactant. Yet, no scientific evidence exists regarding isometric force enhancement in this novel study.

## Introduction

Mint is a well-known natural herb, which grows in most countries with different climates. Peppermint is a hybrid of both spearmint (Mentha Spicata) and water mint (Mentha Aquatica). The peppermint plant contains over 40 distinct chemical compounds (including menthol, menthone, and menthyl acetate) and its consumption safety was proven in toxicological investigations (Nair, 2001 ▶). Peppermint possesses a broad range of biological activities including digestive, choleretic, carminative, antiseptic, antibacterial, antiviral, antispasmodic, antioxidant, anti-inflammatory, myorelaxant, expectorant, analgesic, tonic, and vasodilatator (Duke, et al., 2002 ▶, McKay and Blumberg, 2006 ▶). The main pharmacodynamic effect of peppermint essential oil is related to its dose-dependent antispasmodic effect on the gastrointestinal tract smooth muscles due to the interference of menthol with the movement of calcium across the cell membrane (Grigoleit and Grigoleit, 2005 ▶). 

Previous researches *have* shown a *great impetus* in the search for improvement of athletic performance (Zoladz, et al., 2004 ▶, Meamarbashi and Rajabi, 2013 ▶). Effectiveness of peppermint aroma on perceived physical workload, temporal workload, effort, and anxiety was also studied (Raudenbush, et al., 2002). Other researchers examined the effects of peppermint aroma administered through the nose or orally on the augmenting cognitive performance(Zoladz, et al., 2004 ▶). 

Peppermint aroma caused improvement on tasks related to attentional processes, virtual recognition memory, working memory, and visual-motor response. 

Inhalation of peppermint aroma improved the lung capacity and inhalation ability in healthy participants using a peak flow meter (Raudenbush and Zoladz, 2003 ▶). However, in a four-week randomized, placebo-controlled study on 23 patients with chronic asthma, there were no significant differences in vital capacity, forced expiratory volume, or change in peak expiratory flow rate, between the placebo and the menthol groups (Tamaoki, et al., 1995 ▶). Moreover, previous study on athletic performance, reported that peppermint essential oil had no significant effect on blood oxygen saturation, pulse rate, systolic & diastolic blood pressure, and mean arterial pressure (MAP) (Raudenbush, et al., 2001 ▶). Very recent finding unraveled the effectiveness of ten days oral supplementation with peppermint essential oil on the exercise performance, gas analysis, spirometry parameters, blood pressure, and respiratory rate (Meamarbashi and Rajabi, 2013 ▶).

Hence, the aim of this study was to assess the rapid effects of oral supplementation with peppermint essential oil on the spirometric parameters, visual and audio reaction time, muscular strength tests, and other physiological parameters.

## Materials and Methods

**Participants and study design**

Thirty healthy male university students (24.8±1.26 age; 66.7±4.32 kg body weight; 176.2±4.4 cm height) were randomly selected from 70 volunteers and then randomly divided into experimental and control groups. Ethical approval to conduct this study was obtained from the University Human Ethics Committee. Participants were fully aware of the scope of the study and signed an informed consent form. 

**Methodology**

Participants were familiarized with the laboratory setting and the measurement techniques before data collection. In both groups, maximum grip isometric force, standing vertical jump and standing long jump, forced spirometry test, reaction time tests, blood pressure, heart rates, and breath rate were recorded three times: before, five minutes, and one hour after peppermint essential oil administration. 

**Peppermint essential oil administration**

Participants in the experimental group received 50 µl of pure peppermint essential oil onto the tongue using a sampler and control group similarly received mineral water.

*Handgrip strength*

Dominant hand strength was measured using a computerized handgrip dynamometer with adjustable handle spacing. 

*Standing vertical jump*

Vertical jump test was performed with the knees being bent with their hands on their hips. The difference in distance between the standing reach height and the jump height was recorded. 

*Standing long jump*

The participant stands behind a line marked on the ground with feet slightly apart. A two foot take-off and landing was used, with the swinging of the arms and bending of the knees to provide forward drive. 

*Spirometry test*

After 15 minutes rest and before applying the test, subjects were asked to sit in armed chair. The forced spirometry test was performed using a calibrated handheld electronic turbine spirometer (Microlab spirometer, Micro Medical Limited of Rochester, England) and the best parameters of three forced efforts such as forced vital capacity in first second (FVC_1_), peak expiratory flow rate (PEF), and peak inspiratory flow (PIF) were recorded.

*Reaction time tests*

Two simple reaction time tasks were performed using custom designed reaction time software. In visual task, a yellow box was displayed on the monitor and participant had to press a key as soon as the yellow box appeared. In audio task, participant had to press a key once heard a beep sound. Audio signal and visual presentation had a random variable period (1-3 seconds). These tasks were repeated ten times and the average time was saved in a database. Participants were given a trial practice prior to the actual test and tests were performed in a quiet room. 

*Cardiorespiratory tests*

Blood pressure and heart rate were determined by a digital blood pressure monitor (OMRON One Plus, Omron Healthcare Inc., Bannockburn, IL, USA). Examiner recorded the breath rate during blood pressure measurement.

Statistical analyses

Normal distribution of the data was tested using the Kolmogorov-Smirnov and Shapiro-Wilk tests. Changes in the measures in two groups over time were analyzed using repeated-measures ANOVA with Bonferroni pairwise comparisons. Mauchly's test of sphericity was used to determine whether the assumption of sphericity was being violated by the data and the Greenhouse-Geisser correction was applied when necessary. Measurements before the peppermint supplementation were used as a baseline for the statistical analysis. To calculate the magnitude of the difference between pre-test and post-test, partial Eta squared (partial η^2^) was calculated (where 0.1 is a small, 0.25 is a medium, and 0.4 is a large effect size). All statistical analyses were performed with SPSS 12.0 software (SPSS Inc., Chicago, IL, USA). Difference changes (%) between experimental and control group after five minutes and one hour was compared with before mint administration. Data are presented as means±SD and the level of significance was accepted at p<0.05.

## Results

The Kolmogorov-Smirnov and Shapiro-Wilks tests revealed a normal distribution of the data. Maximum isometric grip force in the experimental group was 17.4% with 27.1% increase while the control group showed 7.5% with 9.0% force decline after five minutes. Overall comparison between experimental and control groups demonstrated 24.9% difference after five minutes and 36.1% difference after one hour (Table 1). The changes in maximum grip force were significant (p<0.05) after one hour of peppermint intake.

Results obtained from standing vertical jump (6.0% and 11.4% vs. 0.0 % and 1.5%) and standing long jump (4.0% and 6.4% vs. -0.4 % and -0.6% ) revealed a considerable increase compared with control group after five minutes and one hour (Table 1). Noticeably, vertical jump increased significantly after five minutes (p<0.05) and one hour (p<0.005) while long jump significantly increased after one hour (p<0.05).

Table 2 shows a significant increase in FVC_1_, PIF, and PEF between experimental and control groups after five minutes of peppermint administration. Only PIF was remained high as compared with baseline after one hour (p<0.5).

Visual reaction time (RT) was significantly decreased after five minutes (10.1%) and one hour (11.8%) following peppermint administration in the experimental group. Audio reaction time showed a significant and shorter reaction time after five minutes (17.1%) and one hour (20.5%) as compared with visual RT in the experimental group (Table 3). 

 Systolic blood pressure as well as heart rate and breath rate were significantly changed after five minutes as compared with baseline. Diastolic BP showed significant changes after one hour as compared with baseline (Table 4). 

**Table 1:** Muscular strength parameters compared between experimental (n=15) and control groups (n=15) during three times of peppermint essential oil administration: before (1), after five minutes (2), and after one hour (3)

**Parameter**	**Group**	**Before (1)**	**After five minutes (2)**	**After one hour (3)**
**Grip test ** **(kg force)**	Experimental	38.2±13.8	44.9±12.6	48.6±11.2 *
	Control	34.2±12.7	31.6±11.0	31.1±12.0
**Standing vertical jump** **(cm)**	Experimental	45.2±5.3	47.9±7.2 *	50.3±7.0 ** ^+^
	Control	45.5±7.2	45.5±7.1	46.2±8.8
**Standing** ** long jump** **(cm)**	Experimental	177.2±22.0	184.2±14.9	188.5±13.7 * ^++^
	Control	173.5±16.0	172.8±15.1	172.5±16.4

Data presented as mean±SD. Statistical analysis used repeated measures with bonferroni pairwise comparison between experimental and control groups during three times: before and after five minutes (1-2), before and after one hour (1-3), and after five minutes and after one hour (2-3). Statistical difference between group 2 and 3 *vs.* group 1

* : p<0.05;

** : p<0.01. Statistical difference between group 2 *vs.* group 3 +: p<0.05; ++: p<0.01.

**Table 2:** Spirometric parameters compared between experimental (n=15) and control groups (n=15) during three times of peppermint essential oil administration: before (1), after five minutes (2), and after one hour (3)

**Parameter**	**Group**	**Before (1)**	**After five minutes (2)**	**After one hour (3)**	
**FVC**_1 _ **(L)**	Experimental	3.98±0.66	4.75±0.57 *		4.84±0.52	
	Control	4.15±0.71	3.87±0.57		3.58±1.09	
**PIF** **(L/s)**	Experimental	304.8±78.1	400.1±85.5 *		446.8±90.5 *	
	Control	290.3±97.0	255.5±91.3		232.8±107.5	
**PEF** **(L/s)**	Experimental	376.4±120.4	505.9±71.08 *		544.5±82.9	
	Control	403.6±101.9	387.6±116.08		321.0±113.8	

Data presented as mean±SD. Statistical analysis used repeated measures with bonferroni pairwise comparison between experimental and control groups during three times: before and after five minutes (1-2), before and after one hour (1-3), and after five min and after one hour (2-3). Statistical difference between group 2 and 3 *vs.* group 1

*: p<0.05. There was not significant difference between group 2 and 3.

**Table 3:** Visual and audio reaction time compared between experimental (n=15) and control groups (n=15) during three times of peppermint essential oil administration: before (1), after five minutes (2), and after one hour (3)

**Parameter**	**Group**	**Before (1)**	**After five minutes (2)**	**After one hour (3)**
**Visual RT** **(msec)**	Experimental	313.1±30	284.4±21 **	280.4±18 *
	Control	321.2±42	313.8±34	318.5±36
**Audio RT** **(msec)**	Experimental	294.0±26	251.2±17 ***	244.1±16 ***
	Control	289.0±22	270.0±27	282.0±42

Data presented as mean±SD. Statistical analysis used repeated measures with bonferroni pairwise comparison between experimental and control groups during three times: before and after five minutes (1-2), before and after one hour (1-3), and after five minutes and after one hour (2-3). Statistical difference between group 2 and 3 *vs.* group 1

* : p<0.05;

**: p<0.01;

*** : p<0.001. There was not significant difference between group 2 and 3.

**Table 4:** Physiological parameters comparison between experimental (n=15) and control groups (n=15) during three times of peppermint essential oil administration: before (1), after five minutes (2), and after one hour (3)

**Parameter**	**Group**	**Before (1)**	**After five minutes (2)**	**After one hour (3)**
**Systolic BP** **(mmHg)**	Experimental	129.9±12.0	131.4±10.7 *	128.2±11.1 ***
	Control	133.0±10.8	122.6±12.7	120.1±9.8
**Diastolic BP** **(mmHg)**	Experimental	74.6±9.2	77.1±8.9	73.0±10.6 ** ^+^
	Control	81.4±7.6	76.8±7.0	74.5±7.1
**Heart rate** **(** **min** ^-1^ **)**	Experimental	68.0±8.5	74.7±6.5 *	71.3±10.5 ^++^
	Control	77.1±7.7	77.3±6.4	73.3±7.1
**Breath rate** **(** **min** ^-1^ **)**	Experimental	17.9±3.2	15.1±3.1 ***	14.6±4.1 ***
	Control	18.6±4.5	18.0±4.3	17.9±4.4

Data presented as mean±SD. Statistical analysis used repeated measures with bonferroni pairwise comparison between experimental and control groups during three times: before and after five minutes (1-2), before and after one hour (1-3), and after five minutes and after one hour (2-3). Statistical difference between group 2 and 3 *vs.* group 1

* : p<0.05;

** : p<0.01;

*** : p<0.001. Statistical difference between group 2 *vs.* group 3 +: p<0.05; ++: p<0.01.

## Discussion

Current results revealed significant improvement in all of the tested variables after five minutes or one hour in the experimental group compared with the control group. Lack of scientific evidence on the effect of peppermint is hindering a comprehensive and detailed treatise regarding a significant increase (36.1%) in the maximum grip force or significant improvement in the standing vertical jump and standing long jump. 

Current research shows an improvement in the experimental group spirometric measurements (FVC_1_, PEF, and PIF) after five minutes (p<0.05). A previous study showed that inhaling peppermint aroma has no effect on the lung function tests and physical performance during acute and intensive exercise (Pournemati, et al., 2009 ▶). A four-week inspiratory and expiratory muscle training program on the respiratory muscle strength and endurance in healthy people has been reported a significant increase in the respiratory muscle strength (Tamaoki, et al., 1995 ▶). It is surprising that in the current study, FVC_1_, PEF, and PIF which are mostly dependent on the strength and speed of shortening of the inspiratory muscles, remarkably improved after five minutes and also one hour of ingestion. 

Menthol (peppermint component) has lowered the surface tension on synthetic surfactant films in an in-vitro study (Zänker, et al., 1980 ▶). The author theorized that it may change the lung surface tension and its function (Zänker, et al., 1980 ▶). The effect of salbutamol as a β_2_-adrenergic receptor agonist and bronchodilator on cycling performance was also investigated (Norris, et al., 1996 ▶) but, there was no significant difference in any variables related to aerobic endurance or cycling performance. In yet another four-week randomized placebo controlled study, 23 participants with chronic mild asthma received either nebulized menthol (10 mg twice a day) or placebo. No effect on the forced expiratory volume was reported in the experimental group (Tamaoki, et al., 1995 ▶). Current findings might be due to the relaxing effect of peppermint on the airway and bronchial smooth muscle tonicity with or without effect on the lung surfactant. 

Current results support the theory of stimulating effect of peppermint on the brain (Umezu, et al., 2001 ▶). In the professional sport competitions, lesser reaction time is crucial in scoring the game. Results obtained in the current study after five minutes and one hour reveal significant and considerable faster visual and audio reaction times. Previous researches have indicated that reaction to the audio signal is faster than reaction to the light (Kosinski, 2012 ▶) and aromas have broad spectrum effects on the human central nervous system (Kobal and Hummel, 1988 ▶, Lorig and Schwartz, 1988 ▶). However the mechanism of action is still unknown but it is hypothesized that the scents of peppermint can stimulate the areas of the brain responsible for alertness (e.g., brain’s reticular activating system) (Raudenbush, et al., 2009 ▶). 

It seems that peppermint has a lowering effect on the heart rate and the systolic blood pressure. Yet in another study, peppermint aroma was administered by nose but no significant effect in both heart rate and blood pressure was observed. Reduction in the arterial smooth muscle tonicity could be a plausible explanation for these effects.

Decrease in the breath rate after five minutes is another challenging finding of this study. The author has previously presented the effectiveness of ten days peppermint essential oil consumption on exercise performance as well as breath rate (Meamarbashi and Rajabi, 2013 ▶). Pondering on the results of pulmonary gas exchange obtained during exercise concurring with lower breath rate may support a theory suggesting the rapid effect of peppermint essential oil on the pneumotaxic center in the brainstem.

Current results imply that only 50 µl of peppermint essential oil ingestion had significant effects on the spirometric measurements (FVC_1_, PIF, and PEF), visual reaction time, audio reaction time, systolic blood pressure, diastolic blood pressure, heart rate, breath rate, grip force, vertical jump test, and long jump test after five minutes and remained effective after one hour. The mechanism underlying the effectiveness of peppermint observed in the current study needs further investigation. 
